# Melittin promotes dexamethasone in the treatment of adjuvant rheumatoid arthritis in rats

**DOI:** 10.3389/fphar.2024.1338432

**Published:** 2024-02-13

**Authors:** Linfu Yang, Xiying He, Dandan Zhi, Yunfei Xue, Xueyang Gong, Kun Dong, Yakai Tian

**Affiliations:** ^1^ Yunnan Provincial Engineering and Research Center for Sustainable Utilization of Honey Bee Resources, Eastern Bee Research Institute, College of Animal Science and Technology, Yunnan Agricultural University, Kunming, China; ^2^ First Clinical Medical College, Yunnan University of Chinese Medicine, Kunming, China

**Keywords:** rheumatoid arthritis, dexamethasone, melittin, curative effect, side effects

## Abstract

**Background:** Rheumatoid arthritis (RA) is an erosive-destructive inflammation of the joints, and the chronic, long-term stiffness and deformation induced by RA are some of the symptoms of arthritis that are difficult to treat. Dexamethasone (DEX) and melittin (MLT) are two interesting anti-inflammatory substances, both of which possess anti-inflammatory effects exerted through the suppression of the immune system. The purpose of this study was to explore the role of MLT in the treatment of RA by DEX as well as to clarify the influence of MLT on the efficacy and side effects of DEX.

**Method:** The rats were injected with Complete Freund’s Adjuvant (CFA) to induce arthritis, followed by treatment with different doses of DEX and/or MLT. The relevant indexes of paw inflammation were determined, and the appetite, growth status, arthritis status, cytokine levels, and organ coefficient of the rats were evaluated. In addition, the paraffin sections of the joint tissues were prepared to analyze the pathological changes.

**Result:** DEX exhibited side effects, notably hindering feed intake and growth, and inducing immune organ lesions in the rats. MLT significantly reduced the side effects of DEX and promoted its efficacy. DEX in combination with MLT demonstrated a synergistic efficacy in RA treatment, showing advantages in detumescence reduction, pro-inflammatory cytokine inhibition, and joint internal pathological improvement.

**Conclusion:** Thus, MLT promoted the efficacy of DEX in adjuvant RA treatment in rats, offering an approach to reduce the use dosage and side effects of DEX.

## 1 Introduction

Rheumatoid arthritis (RA) presents as a chronic, systemic, and autoimmune disease of unknown etiology. Its primary pathological hallmark is joint synovitis, commonly affecting small joints in the hands, wrists, and feet. Early-stage symptoms include joint redness, swelling, heat, pain, and dysfunction of joint movements, while the later stage is often accompanied by stiffness or even deformity to varying degrees and bone and skeletal muscle atrophy. Left uncontrolled, repeated episodes can lead to joint distortion, disability, and permanent loss of mobility (Smolen et al., 2016). RA’s prevalence spans globally, with research indicating its potential association with various factors such as nutrition, metabolism, genetics, occupation, and social environment. Currently, the global prevalence rate of RA stands at approximately 0.5%–1%, and long-term statistical data indicate an increasing trend in prevalence (Deane et al., 2017; Safiri et al., 2019). Furthermore, RA contributes to other complications, such as cardiovascular and neurological diseases, and increases patient mortality, which significantly impacts human health and quality of life (Dougados et al., 2014).

The RA treatment at present continues to use conventional therapeutic drugs or regimens. Nonsteroidal anti-inflammatory drugs, glucocorticoids, disease-modifying antirheumatic drugs (DMARDs; include conventional synthetic DMARDs, biological DMARDs and targeted synthetic DMARDs), and biological agents constitute the primary treatment options for RA. Glucocorticoids, extensively employed as first-line treatment drugs for various autoimmune and inflammatory diseases, are widely used in RA treatment (Abbasi et al., 2018; Prasad et al., 2022; Denis et al., 2023). Dexamethasone (DEX), a pivotal glucocorticoid, significantly alleviates joint pain, stiffness, and swelling associated with RA, remaining a major drug in the market for RA treatment. However, the effectiveness of DEX in treating RA is closely related to its dosage and duration. Achieving a significant therapeutic effect often necessities high doses and long-term administration, which regrettably, leads to severe side effects. For example, prolonged DEX usage can induce hyperadrenocortical syndrome (Cushing syndrome), inhibit the immune system, increase neurological and cardiovascular disease risk, and cause osteoporosis and muscle atrophy, along with other symptoms (Tempark et al., 2010; Oray et al., 2016; Urquiaga and Saag, 2022). Therefore, investigating enhancers to promote DEX’s anti-inflammatory effect, thereby reducing its dosage and mitigating or eliminating its side effects, holds important clinical significance.

Bee venom has been traditionally used to treat RA for centuries and is still employed in various countries and regions. However, due to its complex composition, the components and individual functions within the bee venom remain challenging to decipher, limiting its widespread clinical application (Zhang et al., 2018; Lin and Hsieh, 2020). Fortunately, researchers have successfully isolated melittin (MLT), the primary active ingredient in bee venom, MLT is a natural biological macromolecule composed of 26 amino acid residues, accounting for approximately 40%–60% of the dry weight of bee venom. Previous studies have reported that MLT exerts anti-inflammatory and pathogenic bacteria-killing effects, with many studies reporting the positive effects of MLT in RA treatment. Notably, studies also highlight its anti-cancer potential (Son et al., 2007). In our previous studies, we explored the molecular mechanism of MLT in RA treatment (Yang et al., 2023), which revealed similar anti-inflammatory pathways of MLT and glucocorticoids (Barnes, 2009; Lee and Bae, 2016). Therefore, MLT may serve as a potential glucocorticoid drug enhancer, prompting further exploration into its ability to promote DEX in RA treatment.

This study evaluates the effect of MLT on the anti-RA efficacy of DEX using an *in vivo* experimental model. The purpose is to assess whether MLT can serve as a pharmacodynamic enhancer of DEX for RA treatment, potentially reducing DEX dosage and mitigating its associated side effects. Simultaneously, the study aims to develop new scientific methods for the use of MLT, providing a basis for further basic research and related clinical applications.

## 2 Materials and methods

### 2.1 Agents and chemicals

DEX was obtained from Sinopharm Ronshyn Pharmaceutical Co., LTD (Henan, China), while MLT (purity ≥85%, HPLC) and CFA (containing 1 mg/mL heat-killed *M. tuberculosis*) were obtained from Sigma-Aldrich (Missouri, USA). The ELISA kit for the detection of TNF-α, IL-1β, and IL-6 cytokines was procured from Bioengineering Co., LTD (Shanghai, China).

### 2.2 Animals

Healthy Sprague–Dawley male rats (age: 6–8 weeks old, weight: 200–220 g) were sourced from SPF Biotechnology Co., LTD. (Beijing, China), with license No. SCXK (Jing) 2019–0010. These animals were accommodated in polypropylene cages (55 cm long × 40 cm wide × 20 cm high) with eight rats per cage under standard laboratory conditions. They were fed standard rodent-specific feed and sterile water. A 10-day adaptive feeding period preceded the start of the experiment, allowing the animals to acclimate to the laboratory environment.

The animal experiments were approved by the Life Science Ethics Committee of Yunnan Agricultural University on 30 January 2023 (approval number 202309005) and performed following the National Institutes of Health guidelines for the care and use of laboratory animals.

### 2.3 Induction of RA and grouping

Rats were mildly anesthetized with pentobarbital sodium, and the left hind paw was disinfected with 75% alcohol. A 0.1 mL injection of CFA was administered into the joint cavity (LEE et al., 2005). Rats exhibiting redness, swelling, or erythema in the paw within 1 day were classified as RA rats. These RA rats were randomly divided into five groups (8 rats per group): DEX-h (Dex 0.42 mg/kg); DEX-l (Dex 0.084 mg/kg); DEX-l-MLT (Dex 0.084 mg/kg + MLT 0.1 mg/kg); MLT (MLT 0.1 mg/kg), and RA model group (injected with normal saline). Another eight rats, not injected with CFA, were included in the normal control group (Healthy group). The Healthy group received a matching saline injection when arthritis was induced or drug treatment commenced.

### 2.4 Treatment and inflammation evaluation

Drug treatment was started on the 15th day post-arthritis induction, with a uniform injection volume of 0.05 mL per rat. The required drug concentration for each group was calculated based on the rats’ weights before injection, and the detailed calculation method is provided in the Supplementary Material. The treatment protocol involved a stimulant combined with adaptive administration. For the first three treatments, the drug was injected into the joint cavity of the left hind paw every 2 days, ensuring frequent and continuous exposure to the drug. Considering injection trauma and the animals’ drug tolerance, the last two treatments were administered every 8 days. Daily feed and water intake of each group were recorded by electronic scale and measuring cylinder 2 days before treatment, and the rats were weighed from the beginning of arthritis induction to the end of treatment. The joint thickness was measured every 2 days post-treatment initiation using an electronic vernier caliper, with results reported in millimeters and rounded to two decimal places. The ankle circumference was determined using a flexible tape, and the RA symptoms were scored. The time nodes for measuring the ankle circumference and the scores were found to be consistent with the time nodes for measuring joint thickness. The score was based on the occurrence of inflammation such as erythema and joint swelling: no erythema or swelling of the joint and toes (0 points), swelling of one toe (0.1 points), mild but definite swelling of the joint (0.5 points), or severe swelling (1 point), and the final score is the sum of all toes and joints. Therefore, the score for a given limb ranged from 0 to 1.5 points, while the score for all four limbs ranged from 0 to 6 points (Mossiat et al., 2015). At the end of treatment, the left hind limb of each group of rats was photographed.

### 2.5 Dissected and collected biochemical index samples

Weighing and dissection were performed within 2 h after the last administration. Before dissection, the rats were deeply anesthetized via an intraperitoneal injection of 1–2 mL of 2% sodium pentobarbital (62 mg/kg) according to their body weight (Xia et al., 2023). Subsequently, 3–5 mL of blood sample was collected from the abdominal aorta in vacuum blood collection tubes containing anticoagulant, and the supernatant was obtained by centrifugation at 3,000 rpm for 10 min at 4°C. The collected plasma samples were stored at −80°C for subsequent cytokine content analysis. Three rat organs–liver, spleen, and thymus–were excised, weighed, and organ coefficients were calculated (µ = Y/X, where µ represents organ coefficient; Y represents organ weight; and X represents rat weight) (Bailey et al., 2004). Finally, the joint tissue from the left hind limb was cut and placed in 10% formalin-normal saline fixative, stored at room temperature for 12–24 h, and transferred to an ethylene diamine tetraacetic acid (EDTA) decalcification solution.

### 2.6 Investigation of anti-inflammatory mechanism

The levels of TNF-α, IL-1β, and IL-6 in plasma were determined using a corresponding ELISA kit, following similar procedures for each cytokine. The procedures were performed according to the standard laboratory protocol recommended by the manufacturer. After each of the step reaction between liquids was completed, the absorbance (OD value) was measured at 450 nm wavelength using a microplate reader. Finally, with the concentration of the standard working solution as the horizontal coordinate and OD value as the vertical coordinate, a standard curve was drawn, which was used to determine the concentration of cytokines in the sample and multiplied by the dilution factor when calculating the final concentration.

### 2.7 Histopathological examination

Following decalcification, the joint tissue section (1 mm) was cut longitudinally in the middle of the knee joint in the direction of the wrist bend. Sample sections of each group were consistently obtained from the same part and in the same direction for each group. After dehydrating and removing impurities from the tissues using a graded concentration of alcohol and xylene, a series of conventional paraffin embedding and cutting procedures were performed, yielding sections of 4–5-um thickness. These sections were rehydrated and stained with hematoxylin and eosin (H&E). After staining, the sections were dehydrated and then sealed with neutral gum. The sections were viewed under an optical microscope and photographed, and a 100-fold magnified image of the joint face was used for pathological analysis.

### 2.8 Statistical analysis

Data were analyzed using SPSS 21.0 (IBM) for one-way ANOVA and Tukey’s multiple comparison test, and visualized with GraphPad Prism 9.0 (GraphPad Software, San Diego, California; https://www.graphpad.com/scientific-software/prism/). Except for feed intake and water intake, results were expressed as mean ± the standard error of the mean (SEM), and *p* < 0.05 was considered statistically significant.

## 3 Result

### 3.1 Changes in diet and growth

#### 3.1.1 Feed intake and water intake

**FIGURE 1:**
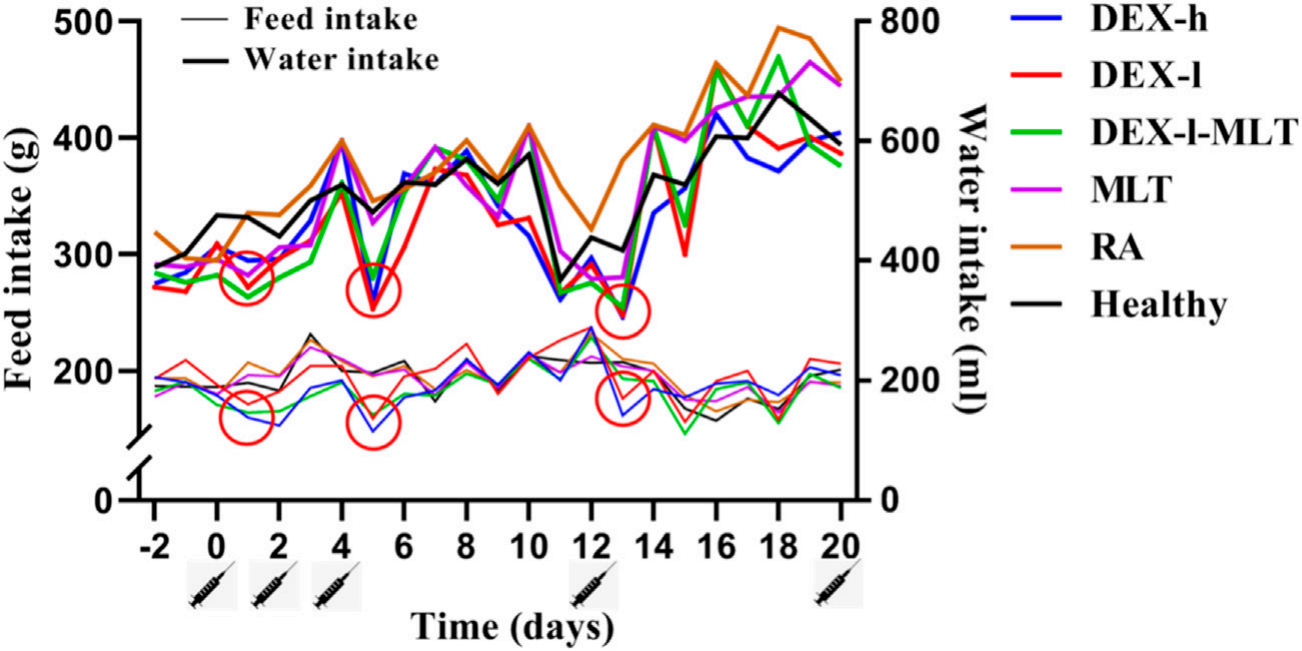
Changes in the feed intake and water intake of rats during treatment. Abbreviations: DEX-h, Dexamethasone - high dose; DEX-l, Dexamethasone - low dose; DEX-l-MLT, Dexamethasone - low dose - melittin; MLT, melittin; RA, rheumatoid arthritis. The location of the marked syringe symbol indicates that the drug was administered at this time, while the location of the red circle indicates the node with significant changes.

Throughout the treatment cycle, the rats displayed significant alteration in feed intake and water intake following drug administration. In the DEX-h, DEX-l, and DEX-l-MLT groups containing DEX, noticeable changes in diet were observed 1 day after treatment, with varying degrees of reduced feed and water intake in three of four analyzed post-administration instances. The inhibitory effect of high-dose DEX was the most significant. Additionally, these three treatment groups exhibited considerable changes and poor stability in feed intake and water intake throughout the whole treatment cycle. Conversely, in MLT, RA, and Healthy groups, the injection of MLT or normal saline did not affect the normal feeding or drinking habits of the rats (Figure 1).

#### 3.1.2 Body weight

**FIGURE 2:**
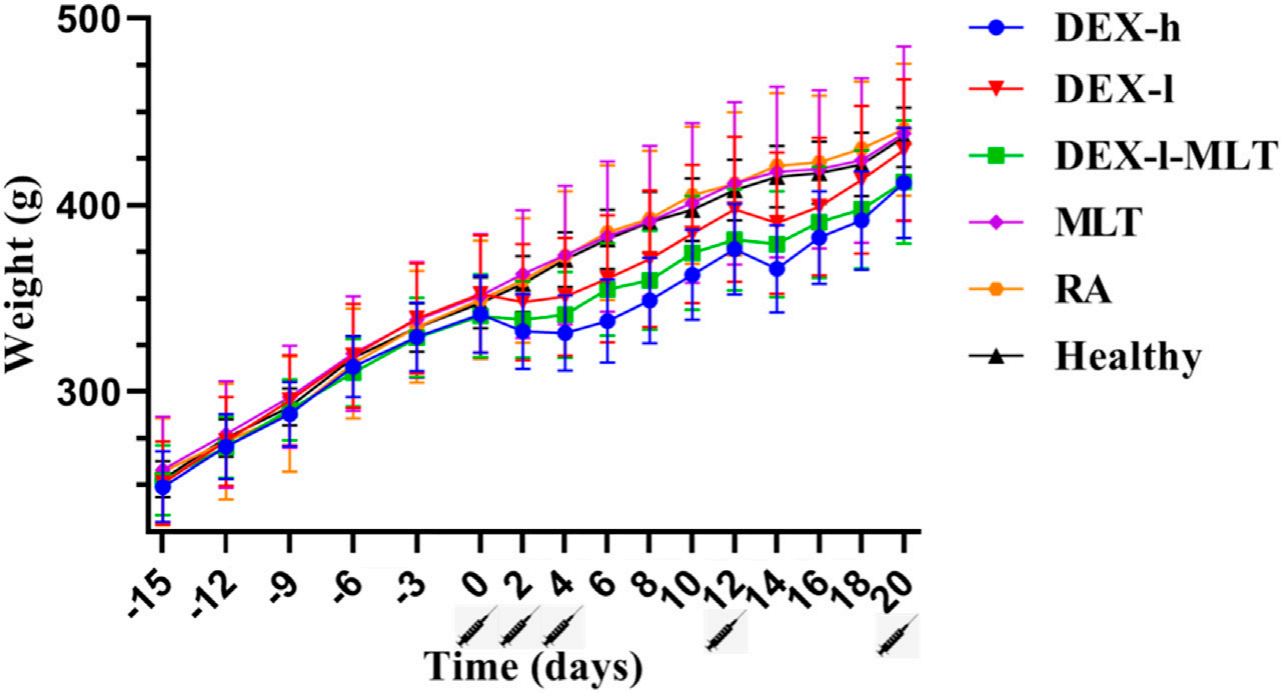
Weight changes in rats from the induction of RA to the end of treatment. Abbreviations: DEX-h, Dexamethasone - high dose; DEX-l, Dexamethasone - low dose; DEX-l-MLT, Dexamethasone - low dose - melittin; MLT, melittin; RA, rheumatoid arthritis. The location of the marked syringe symbol indicates that the drug is administered at this time, and the data is expressed as the mean ± SEM.

Similarly, across the four analyzed post-treatment instances, DEX-h, DEX-l, and DEX-l-MLT groups displayed weight loss or slowed growth in rats. This trend of inhibited weight growth was gradually alleviated with reduced DEX injection doses. The combination of DEX and MLT, while still impacting normal growth compared to the Healthy group, reduced the inhibitory effect of DEX on the growth and development of rats compared with the treatment group receiving the same dose of DEX (Figure 2).

### 3.2 Anti-inflammatory effects of different treatment groups

**FIGURE 3:**
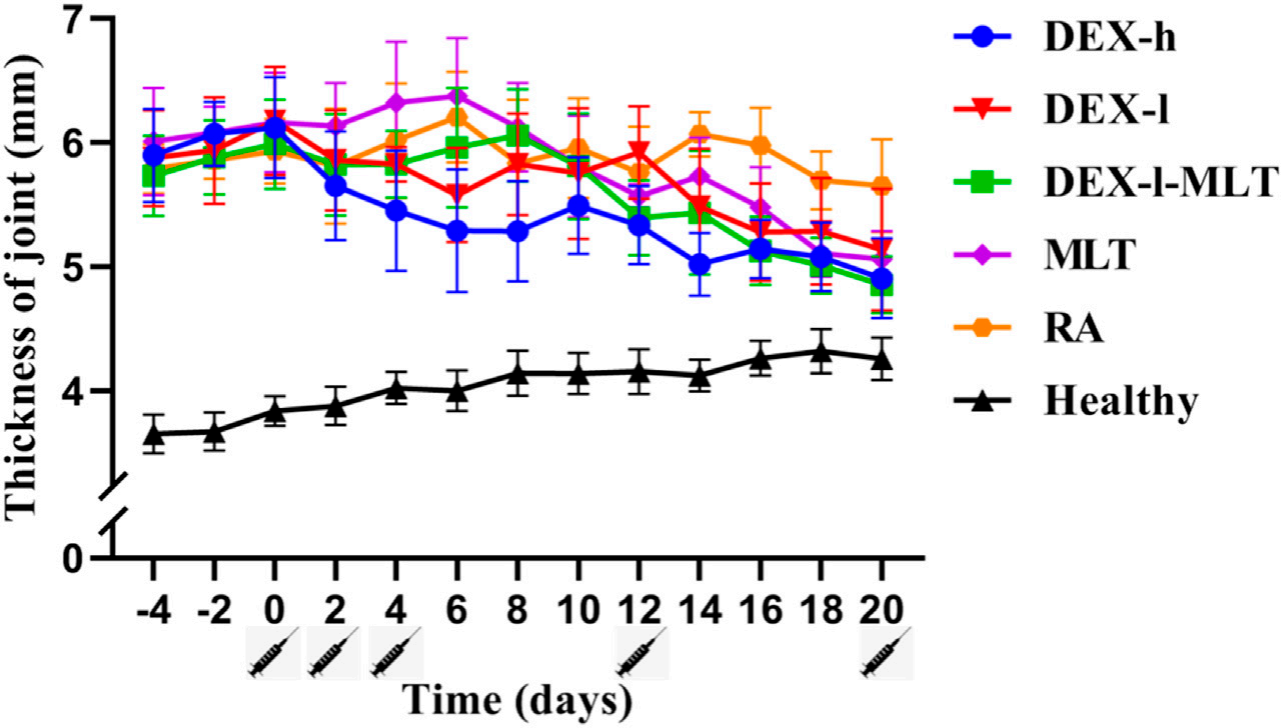
Thickness changes of diseased joints in rats during treatment. Abbreviations: DEX-h, Dexamethasone - high dose; DEX-l, Dexamethasone - low dose; DEX-l-MLT, Dexamethasone - low dose - melittin; MLT, melittin; RA, rheumatoid arthritis. The location of the marked syringe symbol indicates that the drug is administered at this time, and the data is expressed as the mean ± SEM.

The treatment of RA in rats with DEX or MLT produced favorable results. DEX injection alone immediately decreased joint thickness, demonstrating significant detumescence in the DEX-h and DEX-l groups after each injection treatment in a short timeframe. In contrast, the DEX-l-MLT and MLT groups did not exhibit immediate detumescence after each injection but experienced increased joint thickness and exacerbated swelling, with joint thickness beginning to decrease on days 2 and 4 after the third injection and on day 2 after the fourth injection. Notably, the DEX-h, DEX-l, DEX-l-MLT, and MLT treatment groups all demonstrated favorable therapeutic effects in the end, with the DEX-h and DEX-l-MLT groups displaying the least final joint swelling and the most effective therapeutic outcomes (Figure 3).

**FIGURE 4:**
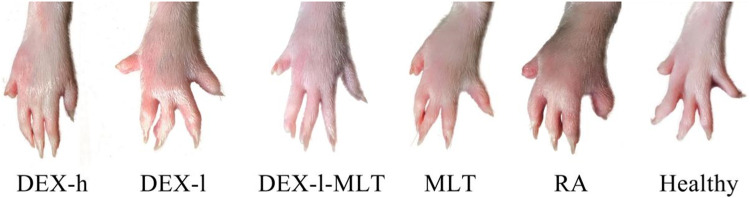
Photographs of diseased paws of rats at the end of treatment (the 20th day). Abbreviations: DEX-h, Dexamethasone - high dose; DEX-l, Dexamethasone - low dose; DEX-l-MLT, Dexamethasone - low dose - melittin; MLT, melittin; RA, rheumatoid arthritis.

**FIGURE 5:**
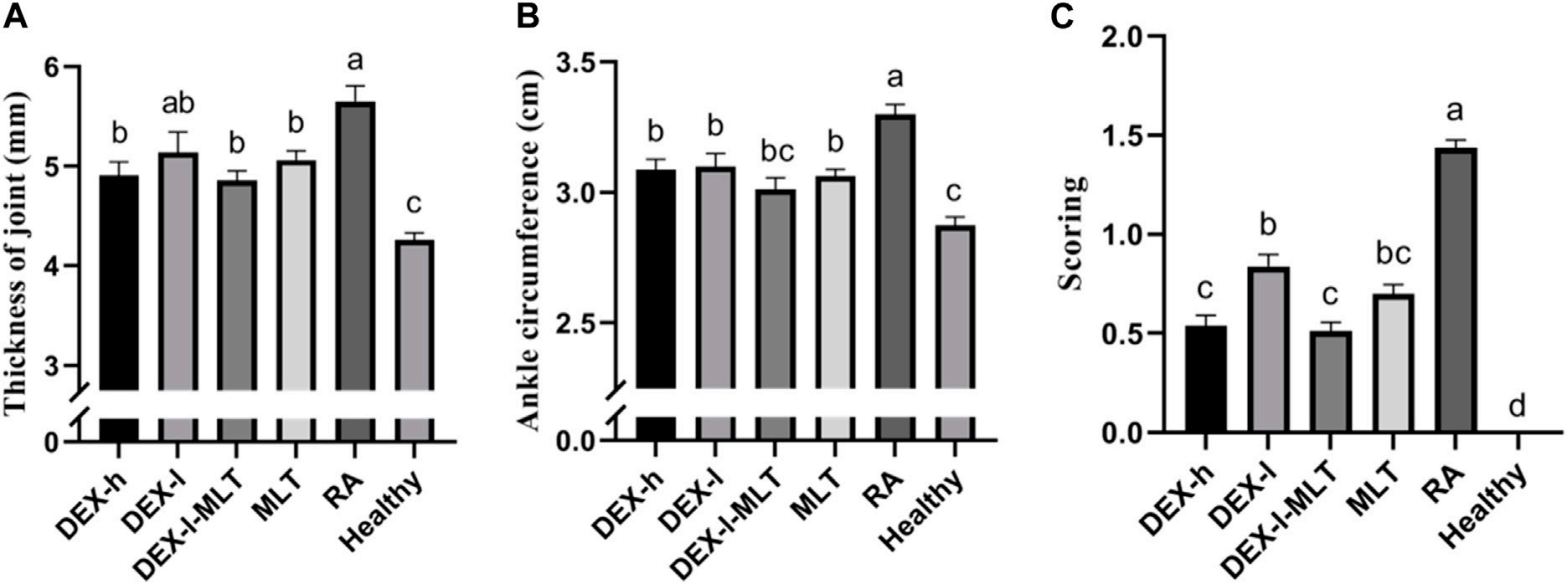
Indicators of inflammation in the diseased joints of rats at the end of treatment, **(A)** joint thickness, **(B)** ankle circumference, **(C)** arthritis score. Abbreviations: DEX-h, Dexamethasone - high dose; DEX-l, Dexamethasone - low dose; DEX-l-MLT, Dexamethasone - low dose - melittin; MLT, melittin; RA, rheumatoid arthritis. The data were expressed as the mean ± SEM, the letters above the bar chart indicated statistical significance, and pairwise comparison showed a significant difference without the same letter (*p* < 0.05), while the same letter showed no significant difference (*p* > 0.05).

At the end of treatment, DEX or MLT demonstrated some anti-inflammatory effects, and different treatments differently improved the extent of erythema and swelling of the paw (Figure 4). The joint thickness in the DEX-h, DEX-l-MLT, and MLT groups significantly decreased compared to the RA group (*p* < 0.05), while no significant difference was observed between the DEX-l and RA groups (*p* > 0.05) (Figure 5A). Ankle circumference and arthritis scores in the DEX-h, DEX-l, DEX-l-MLT, and MLT groups were significantly lower than those of the RA group (*p* < 0.05) (Figures 5B, C). Compared with the Healthy group, most of the three indexes (joint thickness, ankle circumference, and arthritis score) in the above four treatment groups were significantly different (*p* < 0.05). Only the ankle circumference index of the DEX-l-MLT group did not significantly differ from the Healthy group (*p* > 0.05), demonstrating treatment efficacy reaching normal levels (Figure 5B).

### 3.3 Pathological analysis

**FIGURE 6:**
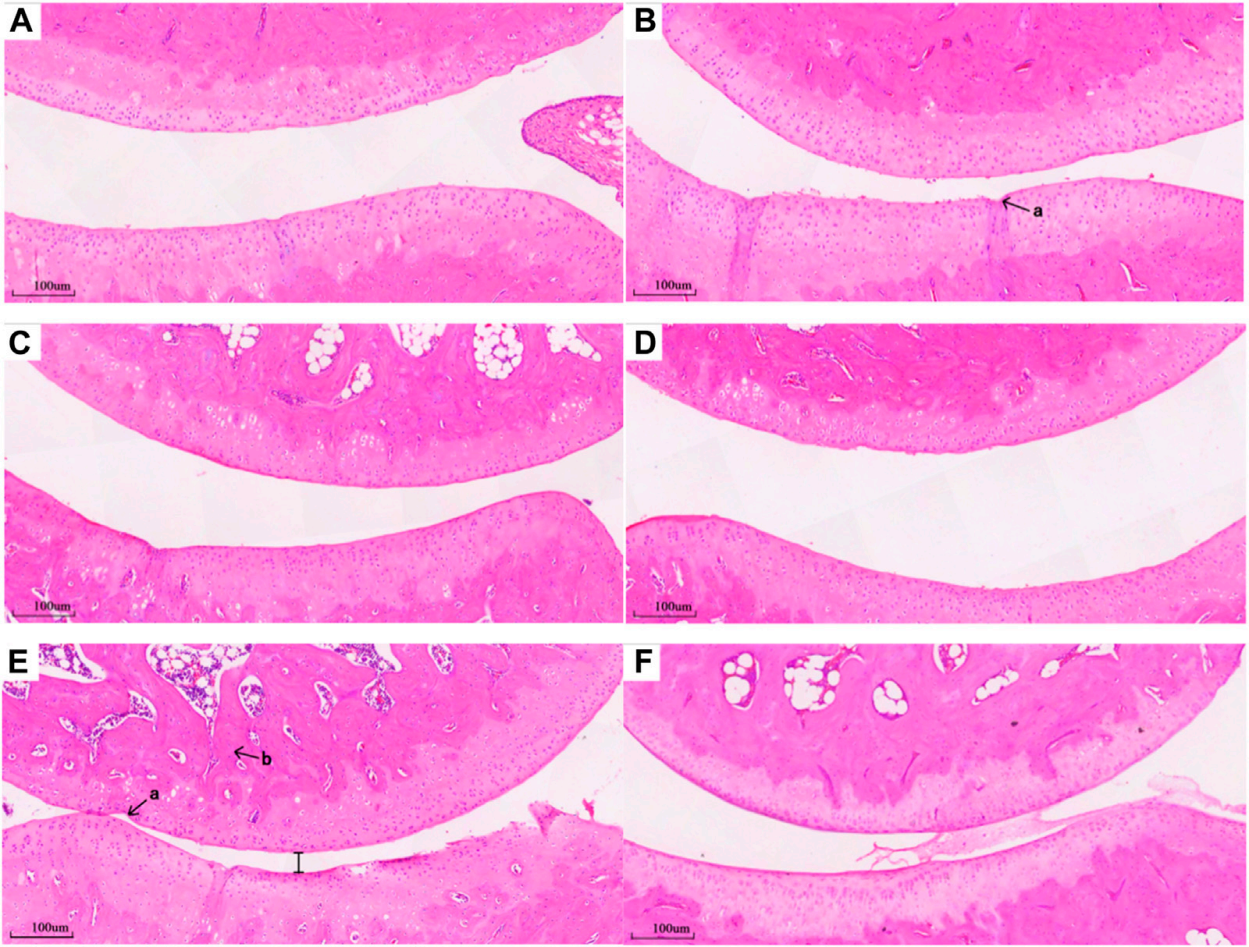
Pathological section of the joint of rats in each group, **(A)** DEX-h, **(B)** DEX-l, **(C)** DEX-l-MLT, **(D)** MLT, **(E)** RA, **(F)** Healthy (×100 magnification). Abbreviations: DEX-h, Dexamethasone - high dose; DEX-l, Dexamethasone - low dose; DEX-l-MLT, Dexamethasone - low dose - melittin; MLT, melittin; RA, rheumatoid arthritis. “a” indicates a gap in cartilage damage, “b” indicates inflammatory cell infiltration.

Compared with the Healthy group (Figure 6F), histopathological examination of the untreated RA group revealed obvious lesions. Microscopic examination revealed a diminished joint space, between the upper and lower bones of the joint, leading to cartilage friction, bone spurs, and inflammatory cell infiltration (Figure 6E). Following DEX or MLT treatment, inflammation was alleviated to varying degrees. Compared with the RA group, inflammatory cell infiltration was reduced, joint space was restored, and cartilage friction lessened, maintaining most of the structure and integrity of normal joints, in the DEX-h, DEX-l-MLT, and MLT groups (Figures 6A, C, D). Compared with the RA group, the DEX-l group showed cartilage thickening and bone density increase, along with an improvement in bone erosion, but residual damage on the cartilage surface remained (Figure 6B).

### 3.4 Levels of TNF-α, IL-1β, and IL-6

**FIGURE 7:**
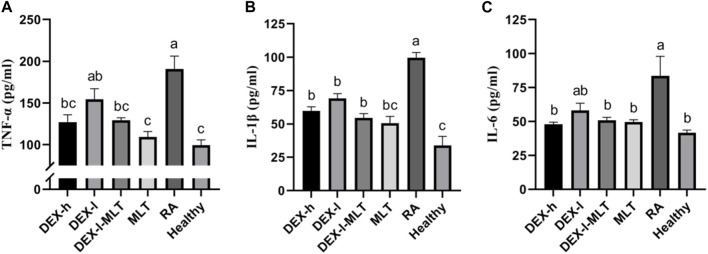
After treatment, the cytokine content of rats in each group, **(A)** TNF-α, **(B)** IL-1β, and **(C)** IL-6. Abbreviations: DEX-h, Dexamethasone - high dose; DEX-l, Dexamethasone - low dose; DEX-l-MLT, Dexamethasone - low dose - melittin; MLT, melittin; RA, rheumatoid arthritis. The data are expressed as the mean ± SEM, the letters above the bar chart indicated statistical significance, and pairwise comparison showed a significant difference without the same letter (*p* < 0.05), while the same letter showed no significant difference (*p* > 0.05).

Among all groups, the levels of TNF-α, IL-1β, and IL-6 cytokines were notably elevated in the RA group, approximately 2–3 times higher than those in the Healthy group. Following treatment with DEX or MLT, the levels of these three cytokines significantly decreased. The most effective treatments were observed in the MLT and DEX-l-MLT groups, and most of them were equivalent to those for the healthy control rats (*p* > 0.05). Although the DEX-l group demonstrated slightly inferior treatment effects compared to other treatment groups, the combination of the same dose of DEX with MLT (DEX-l-MLT group) exhibited cytokine levels equal to or even lower than those in the DEX-h treatment group (*p* > 0.05), indicating significantly enhanced efficacy (Figure 7).

### 3.5 Organ coefficient

**FIGURE 8:**
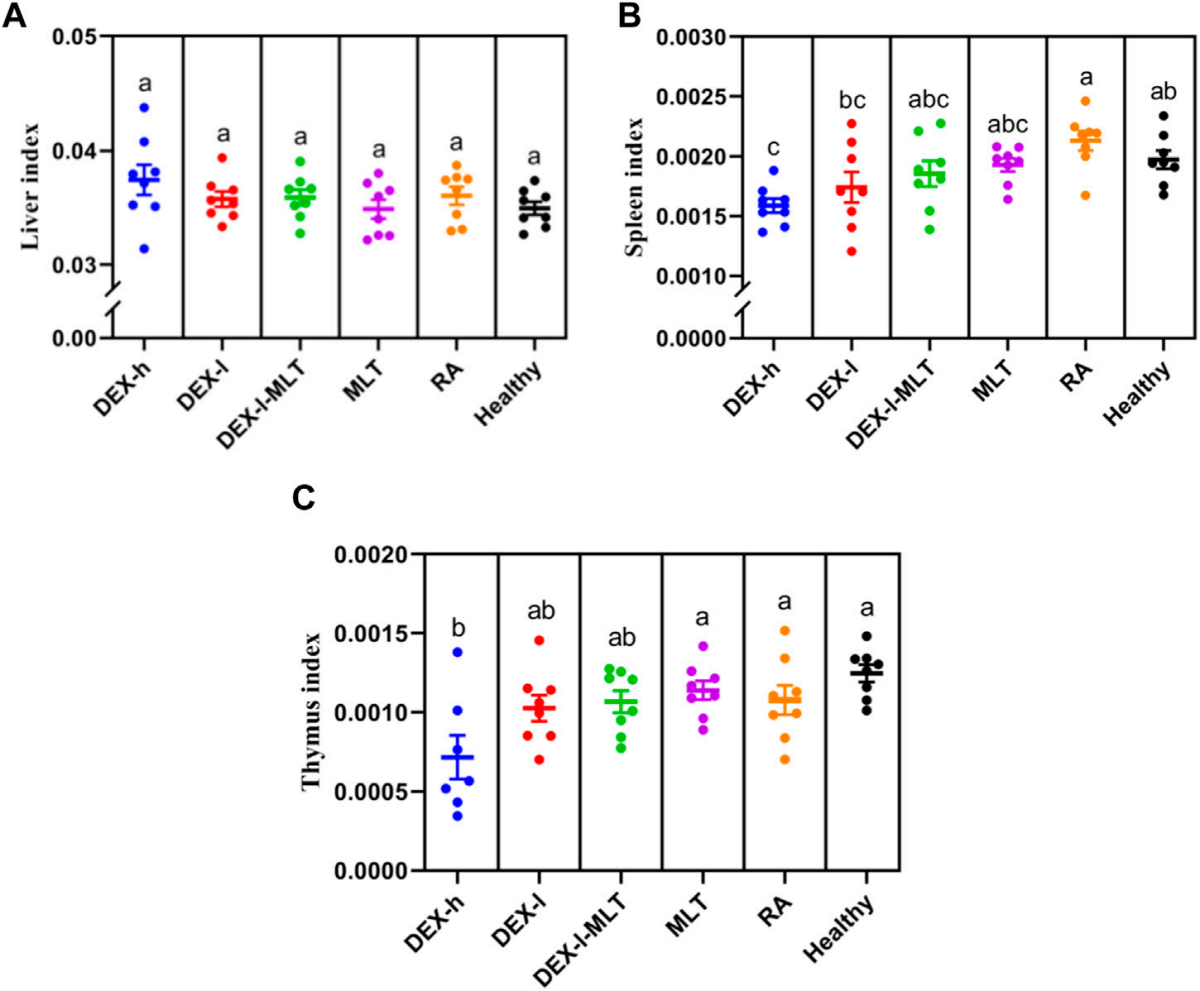
Organ coefficient of rats in each group after the treatment, **(A)** the liver, **(B)** the spleen, **(C)** the thymus. Abbreviations: DEX-h, Dexamethasone - high dose; DEX-l, Dexamethasone - low dose; DEX-l-MLT, Dexamethasone - low dose - melittin; MLT, melittin; RA, rheumatoid arthritis. The data are expressed as the mean ± SEM, the letters above the scatter diagram indicated statistical significance, and pairwise comparison indicated no significant difference without the same letter (*p* < 0.05), while the same letter showed no significant difference (*p* > 0.05).

Liver coefficient variations among all groups (*p* > 0.05) did not display a significant difference. However, the average level in the DEX-h group was higher than in other groups. The individual data distribution in the DEX-h group showed a discrete pattern, while the other groups had closer average levels and more stable overall distributions (Figure 8A). The spleen coefficient in the DEX-h group was notably lower than that in the Healthy group (*p* < 0.05). However, there were no significant differences between the other groups and the Healthy group (*p* > 0.05) (Figure 8B). As the dose of DEX decreased (Figure 8B from left to right), the spleen coefficient became more similar to the Healthy group’s average (except for the RA group). Similar to the spleen coefficient findings, the thymus coefficient in the DEX-h group was significantly lower than that in the Healthy group (*p* < 0.05). The data for the other groups displayed an upward trend from left to right, with no significant differences compared to the Healthy group (*p* > 0.05). Similarly, the data distribution among individuals in the DEX-h and DEX-l groups was discrete in the thymus coefficient diagram (Figure 8C), and an outlier was eliminated from the DEX-h group.

## 4 Discussion

It is known that DEX plays an anti-inflammatory and anti-allergic role by inhibiting the activation of the immune system, which reduces capillary permeability and exudation of inflammatory cells and pro-inflammatory factors (Barnes, 2009; Barnes, 2010). On the other hand, scientists found that MLT interfered with the related inflammatory signaling pathways, promoted cell apoptosis, inhibited immune response, and played anti-inflammatory and antiviral roles (Lee and Bae, 2016). These two compounds share some anti-inflammatory mechanisms. According to a past study, MLT is one of the strongest anti-inflammatory substances known to mankind, and its anti-inflammatory activity is about 100 times that of hydrocortisone and 70 times that of indomethacin (Bava et al., 2023). Notably, MLT exerts a hormone-like effect but has no hormonal adverse effects. Therefore, in this study, we combined these two drugs with the hope of producing an improved therapeutic effect in the treatment of RA.

In this study, the combination of DEX and MLT for RA was compared with the treatment group treated with DEX or MLT alone. We chose to use CFA to develop the RA model rats, as the RA model produced this way resembles the real state and is simple to perform with a high success rate. It has been reported that CFA intervention induces multiple joint inflammation and causes the destruction of articular cartilage after 2–3 weeks; hence, we kept the treatment time as 15 days after the induction (LEE et al., 2005). We selected a dose of 0.1 mg/kg MLT for the treatment, which is the lower limit reported previously, and also because we aimed to generate a stimulative effect (Li et al., 2012). In another study, the authors applied several doses with different concentration gradients, ranging from 0.1 to 1.9 mg/kg, and found the best results with a low dose (0.1 mg/kg) of bee venom (YOU and KWON, 2011).

Hormonal drugs commonly interfere with patients’ normal appetite and mental state, shortly after administration. DEX, known to stimulate the gastrointestinal tract, can lead to loss of appetite, nausea, and vomiting (Noetzlin et al., 2022). In this study, we observed that rats exhibited significantly decreased feed and water intake after DEX injection. Notably, the changes in feed intake and water intake in the groups containing DEX (DEX-h, DEX-l, DEX-l-MLT) were notably unstable throughout the trial period. Therefore, the phenomenon wherein DEX affects the appetite of the test subject has also been confirmed through this experiment. Previous studies have reported the negative effects of DEX on the growth and development of children, especially bone development. When children receive corticosteroid treatment in any way, their growth rate may decrease, which may affect their future height and weight (Gibson et al., 1993; Tack et al., 2016). In this study, the inhibitory effect of DEX on growth and development is obvious. Following DEX injection, the rats displayed slowed growth rate and even negative weight growth, which is particularly detrimental to growing rats. However, MLT treatment for RA in rats did not disrupt the animals’ normal feeding patterns or weight gain trends. Additionally, combining MLT with improved the side effects of DEX. For example, when 0.1 mg/kg of MLT was added to 0.084 mg/kg DEX, the inhibitory effect on feeding and growth in rats was reduced compared to DEX alone.

When oral or injection DEX is used to treat diseases caused by autoimmune inflammation and allergies, it usually takes an hour to take effect (Madamsetty et al., 2022). In contrast, the local swelling caused by bee venom acupuncture therapy or bee stings lasts several hours or even days, varying in duration and intensity among individuals (Cherniack and Govorushko, 2018). In this study, the anti-inflammatory effect of the paw was observed immediately after the injection of DEX. On the other hand, when injected drugs contain MLT, swelling of the paw is always observed after administration, which may be a limitation of the use of MLT or combination drugs. However, the DEX-h, DEX-l, DEX-l-MLT, and MLT treatment groups showed good anti-RA effects in the end, and the efficacy of the DEX-l-MLT group was close to or even greater than that of the DEX-h group. Importantly, DEX content in the DEX-l-MLT group was only 1/5 of that in the DEX-h group. TNF-α, IL-1β, and IL-6 are three typical pro-inflammatory cytokines, and high levels of TNF-α, IL-1β, and IL-6 can be detected in the synovial fluid of patients with RA (Caplazi et al., 2015). These three cytokines activate nuclear factor kappa-B (NF-κB), an important hub of pro-inflammatory gene expression, participate in various inflammatory-related biological pathways, induce a strong immune response in the body, promote the proliferation and differentiation of inflammatory cells, and play a crucial role in the occurrence and development of RA (Hernandez-Rodriguez et al., 2003; Firestein and McInnes, 2017). A previous study similarly demonstrated that *TNF*, *IL1B*, and *IL6* are three important targets involved in the treatment of RA through MLT (Yang et al., 2023). Therefore, the contents of TNF-α, IL-1β, and IL-6 in the plasma of rats in each group were measured in this experiment, revealing higher concentrations in the RA group compared to the Healthy group. After treatment with DEX or MLT, the effect of cytokines in each group was similar to the macro phenotype change trend of paw, except that these three cytokines were most significantly reduced in the MLT group. Moreover, the results of post-treatment cytokine changes were similar to those of two previous studies, one using bee venom therapy for RA and the other fibroblast growth factor 21 (FGF21) combined with DEX for RA (Eltedawy et al., 2020; Sun et al., 2020). Therefore, our results demonstrate the anti-inflammatory potential of MLT. For RA, which is chronic arthralgia, the durability and safety of MLT are matched in terms of prevention and treatment.

The organ coefficient, a critical indicator used in toxicology experiments to gauge animal health, is relatively constant under normal conditions. When an animal is sick, the relevant organs in the body may change their size and weight, effectively changing the organ coefficient (Lazic et al., 2020). Herein, the treatment group was compared with the Healthy control group, wherein the organ coefficient increased, indicating organ congestion, edema or hyperplasia, and hypertrophy. Moreover, the decrease in organ coefficient may indicate organ atrophy and other degenerative changes (Michael et al., 2007; Craig et al., 2014). In this study, three immune-related organs, the liver, spleen, and thymus, were selected for organ coefficient analysis. The three organ coefficients of the DEX-h and DEX-l groups showed a large gap with that of the Healthy group, and the data distribution among individuals was discrete. The spleen coefficient and thymus coefficient of the DEX-h group were significantly different from that of the Healthy group, suggesting that the injection of DEX for RA may cause damage to these three immune organs. Additionally, we also found that there was no significant difference between the three organ coefficients of the treatment group injected with MLT and the Healthy group in this experiment. Compared with the DEX-l group, the DEX-l-MLT group further induced the organs to improve their health status, which was speculated to be caused by the reduction in DEX side effects, owing to MLT addition. As organ weight could vary significantly between individuals, it is challenging to summarize the experimental conclusion by comparing the organ weight of each treatment group. Hence, the organ coefficient transformation value was used for comparison. Additionally, the sampling time to measure the animal organ coefficient is very critical, and it is necessary to avoid sampling when the animal weight changes significantly as it may produce significant differences that do not reflect the real situation (Bailey et al., 2004). In this study, we chose to complete anatomical sampling within 2 h after the last administration. Sampling at this time point could, on the one hand, aid in the real-time monitoring of the drug’s effects on cytokines. On the other hand, the rats at this time had grown for 8 days since the last administration, and the body weight and organ weight of the rats could fully reflect the real changes in organ coefficients, ensuring that our results are robust and reliable. Histopathological examination is a routine technical means to directly reflect the development of the disease. This study focused on several landmark lesions of RA disease, namely, inflammatory cell infiltration degree, cartilage wear, osteoporosis, and fibroarticular capsule status. Among the pathological sections of each group, the above indexes in the DEX-h, DEX-l-MLT, and MLT treatment groups were most similar to those of normal joints, showing better therapeutic effects and further validating the important role of MLT in the treatment of RA with DEX.

This study provides strong evidence that MLT can enhance the anti-inflammatory effect of DEX, effectively promoting the efficacy of DEX and reducing its side effects. Previous research has primarily explored the unilateral anti-inflammatory effects of DEX or MLT alone, but this study combines these two drugs, which are completely different, fully considering their synergistic effect, and has unveiled novel discoveries. However, the exploration of other aspects of this experiment is limited, warranting further study to explore the interaction and mechanism of the two drugs.

## 5 Conclusion

This study underscores the positive role of MLT in the treatment of RA with DEX. MLT promotes the efficacy of DEX in the treatment of RA, effectively enhancing DEX’s therapeutic effects and substantially reducing the required dosage. At the molecular level, the combination of drugs significantly reduced the contents of three pro-inflammatory cytokines, namely, TNF-α, IL-1β, and IL-6, and effectively improved the pathological status of the affected joints. Furthermore, MLT effectively alleviated the side effects induced by DEX, such as reduced appetite, inhibited growth and development, and impaired immune organs. Thus, the combination of MLT and DEX in the treatment of RA is a potential and promising therapy, providing a new strategy for its application. In the future, it will be necessary to design detailed experimental protocols to complement the internal response of MLT in DEX therapy.

## Data Availability

The datasets presented in this study can be found in online repositories. The names of the repository/repositories and accession number(s) can be found below: https://www.jianguoyun.com/p/DTG0MOMQy-iTDBiXq6QFIAA.
