# Supplementary material for: Melittin promotes dexamethasone in the treatment of adjuvant rheumatoid arthritis in rats
Source: Front Pharmacol. 2024 Feb 13;15:1338432. doi: 10.3389/fphar.2024.1338432 (PMC10896847; doi:10.3389/fphar.2024.1338432)
Supplement: Supplementary file 1 [file Table1.DOCX]

**Supplementary material**

Using a 300g rat as an example, the required concentration of DEX or MLT was calculated, and so on for other weight rats.

**Table 1.** Detailed injection protocols for different treatment groups.

| Group | Rat body weight | injected dose | agent concentration |
| --- | --- | --- | --- |
| DEX-h(Dex0.42mg/kg) | 300g | 0.05ml | 2.52mg/ml DEX |
| DEX-l (Dex0.084mg/kg) | 300g | 0.05ml | 0.504mg/ml DEX |
| DEX-l-MLT (Dex 0.084mg/kg + Mlt 0.1mg/kg） | 300g | 0.05ml | The solution contains both 0.504mg/ml DEX and 0.6mg/ml MLT |
| MLT（Mlt 0.1mg/kg） | 300g | 0.05ml | 0.6mg/ml MLT |
